# Silicon photonic modulators with a 2 × 1 Fabry–Perot cavity

**DOI:** 10.1515/nanoph-2024-0488

**Published:** 2025-01-14

**Authors:** Hengzhen Cao, Jin Xie, Weichao Sun, Mingyu Zhu, Yuluan Xiang, Gong Zhang, Jingshu Guo, Yaocheng Shi, Daoxin Dai

**Affiliations:** State Key Laboratory of Extreme Photonics and Instrumentation, College of Optical Science and Engineering, International Research Center for Advanced Photonics, Zhejiang University, Zijingang Campus, Hangzhou 310058, China; Jiaxing Key Laboratory of Photonic Sensing & Intelligent Imaging, Intelligent Optics &Photonics Research Center, Jiaxing Research Institute, Zhejiang University, Jiaxing 314000, China; Intelligent Optics and Photonics Research Center, Jiaxing Research Institute, Zhejiang University, Jiaxing 314000, China; Ningbo Research Institute, Zhejiang University, Ningbo 315100, China

**Keywords:** modulator, silicon photonics, silicon modulator, resonant cavity, Fabry–Perot cavity

## Abstract

Silicon photonics modulators based on a 2 × 1 Fabry–Perot (FP) cavity, which is circulator-free, are proposed and demonstrated by introducing two asymmetric multimode-waveguide grating (AMWG) reflectors and a short straight modulation section with interleaved PN junctions. In particular, the straight modulation section in the FP cavity is broadened to be far beyond the single-mode regime, alleviating the inherent sensitivity to the variations of waveguide dimensions and thus reducing stochastic resonance-wavelength variations. The *Q* factor of the FP cavity is manipulated by optimally manipulating the reflection of the AMWGs, and the modulation bandwidth is enhanced to be over 40 GHz by utilizing the optical peaking enhancement effect, which happens when operating at the wavelength slightly detuning to its resonance wavelength. Eye diagrams for high-speed modulation with 50 Gbps are also demonstrated in experiments. Finally, wafer-level measurement is conducted by characterizing the silicon photonic modulators based on the 2 × 1 FP cavity and a conventional microring fabricated on the same chip, experimentally revealing an average improvement of 43 % in minimizing the random resonance-wavelength variation, which is attributed to the implementation of broadening the straight modulation section in the FP cavity.

## 1 Introduction

The burgeoning demand for high-speed data transmission across optical networks has markedly intensified research interest. Consequently, the development of high-performance optical transceivers has become imperative. Among various integrated photonic technologies, silicon photonics distinguishes itself with unique advantages, including compatibility with complementary metal–oxide–semiconductor (CMOS) processes, cost-effectiveness, and superior integration density [1], [2], [3]. High-speed electro-optic (EO) modulators, which are critical devices in optical transceivers, have undergone extensive development in recent decades [4], [5]. Silicon photonic modulators, leveraging the free-carrier plasma dispersion effect, exhibit exceptional potential and performance, heralding them as a frontrunner for the monolithic integration of optical transmitters on silicon [6], [7].

Owing to the inherently weak electro-optic effect in silicon, long modulation regions are frequently required. Consequently, Mach–Zehnder modulators (MZM) featuring traveling-wave electrodes have been engineered to enhance the modulation efficiency and bandwidth, utilizing either differential drives [8], [9], [10] with coplanar waveguides or single-drive push-pull structures [11], [12], [13], [14]. In Ref. [9], a 3-mm-long differential-drive MZM was proposed with a bandwidth of 30 GHz and a modulation efficiency of 1.5 V cm. In Ref. [12], a 4.2-mm-long single-drive push-pull MZM was demonstrated with a 35-GHz bandwidth and 112-Gbps PAM4 signals. Additionally, T-shaped electrodes have been validated to provide flexibility in matching the group velocity and the impedance, thus enhancing the bandwidth [15], [16]. As illustrated in Ref. [15], a 4.25-mm-long MZM was demonstrated to achieve a bandwidth of up to 41 GHz. Recently, the integration of a segmented-electrode silicon photonic modulator with a distributed driver has been advanced as a promising approach to resolve the trade-off between expansive bandwidths and high modulation extinction ratios in optical transmitters [17], [18]. Although traveling-wave MZMs are predominantly utilized in optical transmitters for their high reliability and broad optical bandwidth, their suitability for high-density, multichannel high baud-rate applications is compromised by their substantial footprints and elevated static power consumption.

Alternatively, innovations in slow-light [19], [20], [21], [22] and folded-waveguide [23], [24], [25] structures have been showcased recently. For instance, slow-light silicon photonic modulators, typically as short as 0.57 mm, feature a bandwidth of approximately 42 GHz, albeit with a limited optical bandwidth of 3.3 nm [22]. For the folded-waveguide structures, the phase-shifter section is shortened at the sacrifice of the electro-optic bandwidth reduction due to small RC-limited bandwidth, which was reported to be merely 17 GHz [24]. Microring modulators (MRMs) have also emerged as a promising alternative for compact footprints, widely demonstrated over the past decades [26], [27], [28], [29], [30]. In recent years, MRMs have gained popularity as a choice for silicon photonic modulators, attributed to their structural simplicity and design convenience. For instance, a compact MRM measuring 62.8 μm in length, with a bending radius of 10 μm, achieved a 50 GHz electro-optic bandwidth and a modulation efficiency of 0.52 V cm [30]. The wavelength selectivity of MRMs proves advantageous for WDM systems, as they facilitate cascading to modulate and multiplex multiple data channels concurrently [31], [32], [33]. This approach enables the realization of optical transmitter chips with ultra-compact footprints and minimal power consumption. Consequently, this has heightened the appeal of MRMs in recent years. For such MRMs, it is customary to select 450-nm-wide single-mode silicon ridge waveguides in the modulation regions to prevent the excitation of higher-order modes. Here, the bending radius is typically set at greater than 8 μm to minimize bending losses. It is noteworthy that cavity-based optical modulators generally exhibit a narrow optical bandwidth. Consequently, precise alignment of the working wavelength is crucial, given the sensitivity of the resonance wavelength to minute, random dimensional variations in the fabricated microring waveguide. Therefore, the use of MRMs often necessitates meticulous thermal tuning and stringent control [34], [35], [36], which elevates system complexity and augments power consumption, thus limiting their widespread application in systems. Thus, there remains an urgent need to develop a novel silicon photonic modulator based on a new cavity design, wherein the resonance wavelengths are less sensitive to the dimensional variations of the cavity waveguide, facilitating easier alignment of the working wavelength.

It is worth noting that another favored approach involves utilizing an FP cavity to construct a silicon photonic modulator, which includes two reflectors and a modulation region. However, the modulated light is reflected to the input port for conventional FP cavities [37]. Consequently, this necessitates the inclusion of an additional optical circulator to separate the input and reflected light, thereby complicating the system and hindering further monolithic integration. We have recently introduced an innovative 2 × 2 FP cavity, incorporating specially designed asymmetric multimode waveguide gratings (AMWGs) as substitutes for traditional Bragg gratings. These novel 2 × 2 FP cavities have facilitated the development of high-performance lithium-niobate-on-insulator (LNOI) optical modulators [38], [39], offering considerable advantages by circumventing the use of bending structures, which matches well with the requirements of LNOI photonics.

In this paper, we introduce silicon photonic modulators utilizing a 2 × 1 FP cavity, which operate effectively without an optical circulator, facilitated by asymmetric AMWGs and a mode (de)multiplexer. Unlike MRMs, the FP-cavity modulator (FPM) features a shortened 30-μm-long modulation region, because no bend is included in the cavity. Crucially, the modulation section of the proposed FPM is expanded to be 1-μm-width and is well beyond the single-mode regime [40], significantly diminishing the sensitivity of the resonance wavelength to the random dimensional variations. In addition, we incorporate interleaved-doping regions within the modulation region to enhance the modulation efficiency, while it can be fabricated with a standard multiproject wafer (MPW) foundry of silicon photonics. For the fabricated FPM, the measured modulation efficiency is 0.62 V cm and the measured 3-dB bandwidth is up to 36 GHz when designed with the optimal *Q* factors by choosing different period numbers of AMWGs. Notably, further optimally detuning the working wavelength and reducing the *Q* factor, the 3-dB bandwidth can be enhanced to exceed 40 GHz [41]. The eye diagrams of the modulated NRZ signals with bit rates of 20, 30, 40, and 50 Gbps are also demonstrated. Finally, the FPMs on the 25 chips from the same wafer were measured, showing a 43 % lower than MRMs in random variation of the resonance-wavelength shifts, which experimentally verifies the superior wavelength stability achieved with the present FPMs.

## 2 Structure and design

**Figure 1:**
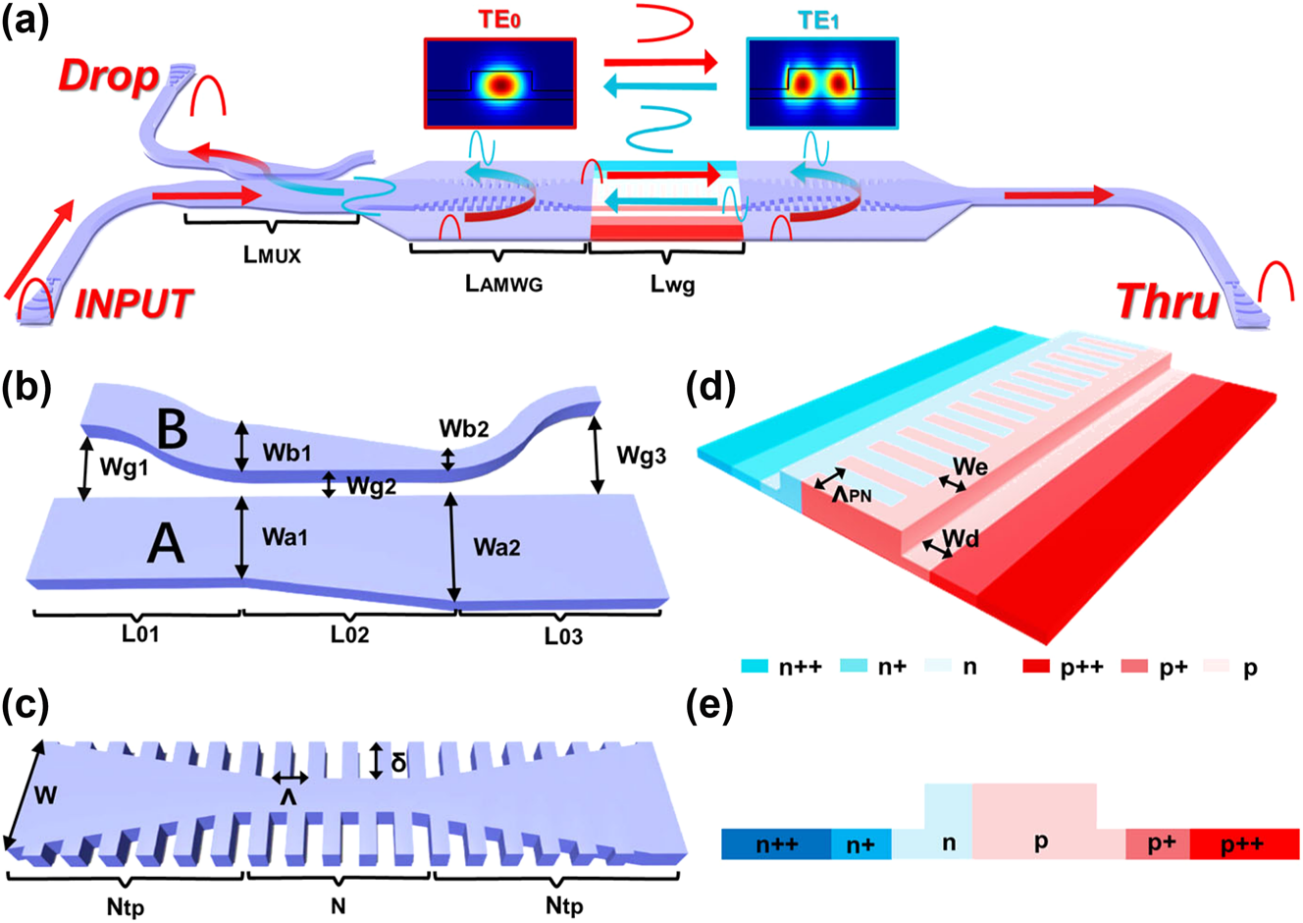
Schematic of the (a) FP-cavity silicon modulator; (b) adiabatic dual-core tapered mode (de)multiplexer; (c) asymmetric multimode-waveguide gratings (AMWGs); (d) novel interleaved PN doping; and (e) cross section of the active PN doping profile.

As depicted in Figure 1(a), the schematic structure of the proposed FPM consists of a TE_0_/TE_1_ mode (de)multiplexer (see Figure 1(b)), two AMWGs serving as the mirrors of the FP cavity (see Figure 1(c)), and interleaved PN junctions (see Figure 1(d) and (e)). The input strip waveguide and the rib waveguide utilized in the FP cavity were connected by the introduction of bi-level tapered waveguides. The rib waveguide has been etched with a depth of 130 nm to construct the slab. The AMWGs were designed with a large reflection bandwidth and partially reflect the forward TE_0_ (TE_1_) mode to the backward TE_1_ (TE_0_) mode when the wavelength is within the reflection bandwidth. When the wavelength of the input TE_0_ mode is within the reflection bandwidth, both the resonance and the mode conversion (TE_0_–TE_1_, TE_1_–TE_0_) in the FP cavity occur accordingly. Subsequently, the backward TE_1_ mode is converted to the TE_0_ mode by the mode (de)multiplexer and output from the Drop port in Figure 1(a). The separation of the input and reflected light obviates the need for an external optical circulator in the FP-cavity modulator. Meanwhile, the forward TE_0_ mode traverses the FP cavity and output from the Thru port in Figure 1(a). The fundamental operating principle of the FP cavity modulator is utilizing the electrical tuning of the resonance wavelength to facilitate on-off modulation. Variations in the effective refractive index, induced by the free carrier dispersion effect in the active region for the phase shifter, result in a slight resonance wavelength shift. An interleaved doping integrating both lateral and conventional interleaved p-n junctions [42] is introduced to concurrently enhance the modulation efficiency for both TE_0_ and TE_1_ modes. To minimize the random variation of the resonance wavelength due to the random fabrication errors, the width *W* of the FP-cavity waveguide is chosen as wide as 1 μm, which is far beyond the single-mode regime and is hardly applicable for microring modulators.

### 2.1 Mode (de)multiplexer

Figure 1(b) shows the schematic of the mode (de)multiplexer, which incorporates an adiabatic dual-core tapered coupler for large fabrication tolerance and wavelength-insensitive operation. The operational principle of the mode (de)multiplexer relies on the adiabatic evolution of supermodes, as discussed in Ref. [43]. The details for designing the mode (de) multiplexer can be referred to in Supplementary Material 1. In our design, the widths of the two waveguides are configured to (*w*_a1_, *w*_b1_) = (0.42, 0.28) μm and (*w*_a2_, *w*_b2_) = (0.6, 0.12) μm. The gap widths *w*_g1_, *w*_g2_, and *w*_g3_ have been selected as 1.5, 0.18, and 1.8 μm, respectively, with the taper lengths *L*_01_, *L*_02_, and *L*_03_ determined as 20, 35, and 15 μm, respectively.

### 2.2 AMWG and FP cavity

**Figure 2:**
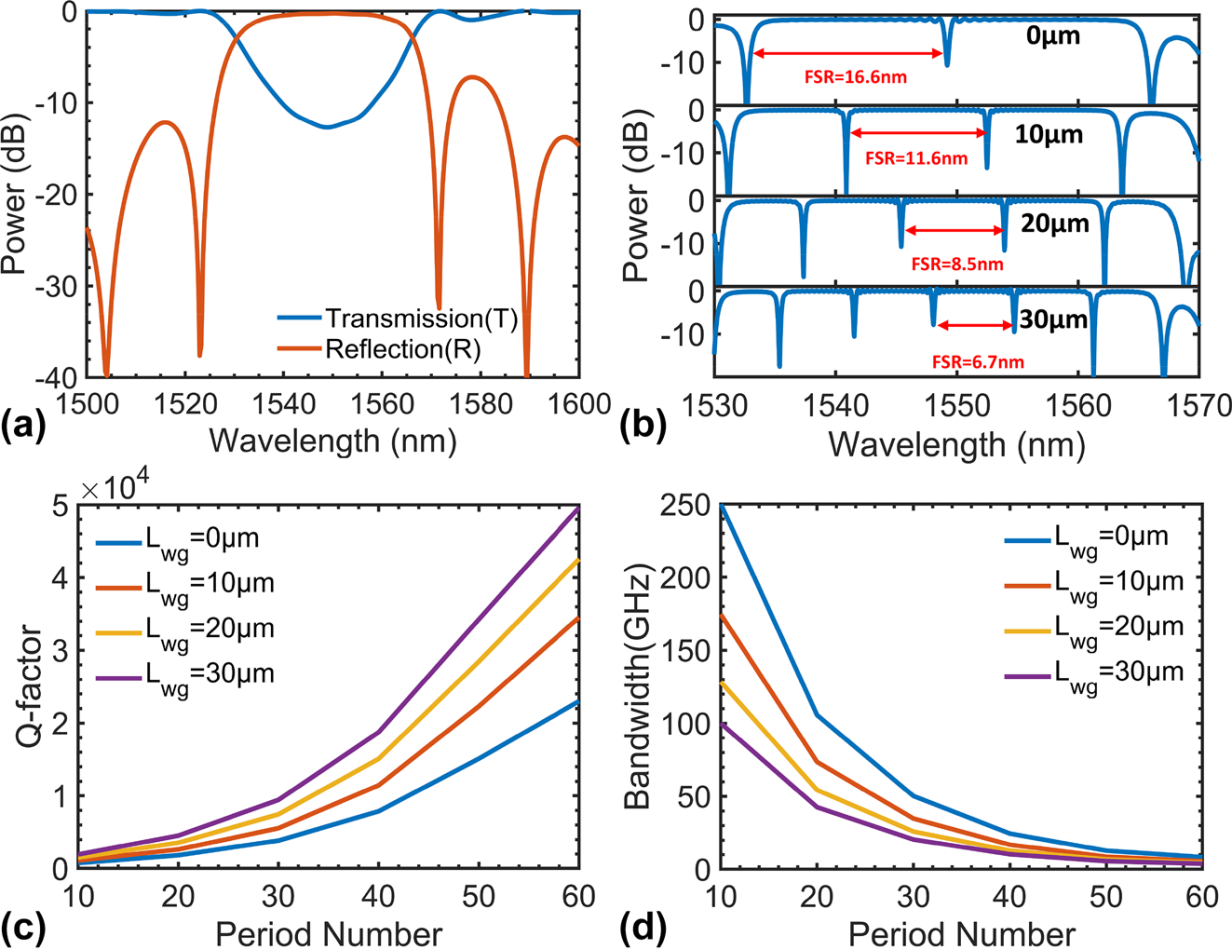
The simulated results for the FP-cavity modulators. (a) The calculated transmission (*T*) and reflection (*R*) of the AMWG for the launched TE0 mode when the period number *N* = 40; (b) the simulated resonant spectrum of the FP cavity silicon modulator under different *L*_wg_; the simulated (c) *Q* factor and the corresponding *Q*-factor-limited bandwidth under different period numbers when *L*_wg_ = 0, 10, 20, and 30 μm, respectively.

Figure 1(c) shows the schematic configuration of the proposed AMWG. Unlike the traditional Bragg gratings, an asymmetric wide waveguide featuring a half-period offset between two sidewall gratings facilitates the conversion of the input TE_0_ (TE_1_) mode into the reflective output TE_1_ (TE_0_) mode when the wavelength falls within the Bragg reflection bandwidth. The designed Bragg wavelength *λ*_*B*_ is determined as *n*_eff0_ + *n*_eff1_ = *λ*_B_/Λ, where *n*_eff0_ and *n*_eff1_ represent the effective refractive indices of the TE_0_ and TE_1_ modes, respectively, and Λ is the grating period. The rib waveguide with a 90-nm-thick slab was selected to accommodate the necessary electrical connections and carrier transitions in the FP cavity modulator. To mitigate unwanted reflection losses at the front and back ends of the grating section caused by abrupt changes in the effective refractive index, adiabatic grating tapers are employed. Here, the corrugation depth *δ* of the gratings varies linearly from zero to the maximum to connect the straight waveguide and the Bragg grating waveguide. The period number *N*_tp_ of the tapered Bragg grating is optimized to be 20 for low reflection loss. The corrugation depth is optimized to be large enough to ensure a broad reflection bandwidth. The total length of the AMWG is determined to be *L*_AMWG_ = (2*N*_tp_ + *N*)Λ, where *N* is the period number of the Bragg grating in the middle. In our design, the corrugation depth is 180 nm with a corresponding grating period Λ of 306 nm to achieve a broad Bragg bandwidth around the centered wavelength of 1,550 nm. Figure 2(a) shows the calculated transmission (*T*) and reflection (*R*) of the designed AMWG for the launched TE_0_ mode when the period number *N* = 40, where a simulated large reflective 3 dB bandwidth of approximately 35 nm is achieved. A prominent sidelobe is observed due to the absence of longitudinal apodization in our AMWG design. To explore the resonance characteristic of the proposed FP cavity modulator, the length *L*_wg_ of the straight section between the two AMWGs (i.e., the phase shifter region) is varied as 0, 10, 20, and 30 μm. Figure 2(b) shows the simulated resonant spectrum at the drop port for the cases with different *L*_wg_ and the same period number of 40. The free spectral range (FSR) decreases as the cavity length increases, and the maximum FSR of 16.6 nm is observed when *L*_wg_ = 0 and is mainly constrained by the introduction of the tapered grating. On the other hand, when increasing the length *L*_wg_ of the straight section whose width is broadened to be beyond the single-mode regime, the random variation of the resonance wavelength of the FP cavity can be reduced. As a trade-off, we choose the length *L*_wg_ to be 30 μm, and the corresponding FSR is around 6.7 nm.

In conventional resonant modulators, the modulation bandwidth is constrained by the RC time constant and the photon lifetime within the cavity. Regarding the compactness of the cavity, the *Q* factor often exerts a critical influence over the modulation bandwidth. For FP cavities, the *Q* factor is primarily constrained by the AMWGs’ reflectivity, which can be designed flexibly by adjusting the period number of the AMWG. In order to employ a moderate *Q* factor in the FP-cavity modulator for balancing the bandwidth and the modulation extinction ratio, it is imperative to choose the period number appropriately. Figure 2(c) shows the simulated *Q*-factor for the cases with different *L*_wg_ as the period number increases and the corresponding modulation bandwidth is given in Figure 2(d). As it can be seen, the *Q* factor increases with the cavity length as well as the period number. In the present case, the *Q* factor increases from 2,000 to 48,000 (when *L*_*wg*_ = 30 μm) as the period number increases from 10 to 60 and the corresponding modulation bandwidth constrained by the *Q* factor decreases from 100 GHz to 4 GHz. Finally, the FP modulator is designed with a *Q* factor of 9,400 and a finesse of 40.6.

### 2.3 Tolerance analysis

**Figure 3:**
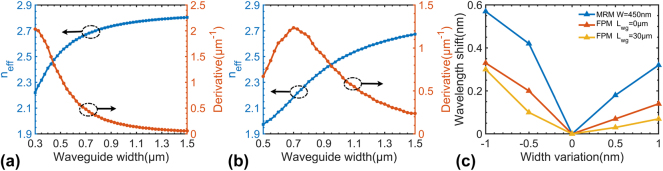
Calculated effective refractive index and its derivative versus waveguide width for (a) TE_0_ and (b) TE_1_ mode; (c) simulated resonant-wavelength variation under ±0.5 and ±1 nm waveguide width variation for MRM with a 450-nm-wide waveguide and FPM with 1-μm-wide waveguide.

Although resonant modulators benefit from compact footprints, their sensitivity to fabrication variations necessitates substantial thermal tuning power to manage the resonance wavelength, thereby leading to increased power consumption. A viable approach is utilizing multimode photonics technology, particularly with optical waveguides broadened far beyond the single-mode regime [44]. For optical resonators, the resonance wavelength shift is dependent on the effective refractive index deviation *δn*_eff_ arising from the variation of the waveguide dimensions (Δ*W* and Δ*H*) and one has:

$$\delta n_{\text{eff}}=\frac{\partial n_{\text{eff}}}{\partial W}\Delta W+\frac{\partial n_{\text{eff}}}{\partial H}\Delta H$$

where ∂*n*_eff_/∂*H* and ∂*n*_eff_/∂*W* are the derivatives of the effective refractive index versus thickness and width, respectively. For 220-nm-thick SOI waveguides, ∂*n*_eff_/∂*H* is a constant around 3.75 μm^−1^ and the thickness’s random variation is negligible within a small area. Consequently, we concern more to minimize the coefficient ∂*n*_eff_/∂*W* to greatly reduce the deviation *δn*_eff_ due to the variation Δ*W*. Figure 3(a) and (b) show the calculation results for *n*_eff_ and ∂*n*_eff_/∂*W* for both TE_0_ and TE_1_ modes in the rib waveguide as the core width varies, clearly demonstrating that ∂*n*_eff_/∂*W* decreases rapidly when the waveguide is broadened to be far beyond the single-mode regime (e.g., *W* > 900 nm). For example, one has ∂*n*_eff_/∂*W* as low as 0.2 μm^−1^ for the TE_0_ mode when *W* = 1 μm. In contrast, for the 0.45-μm-wide optical waveguide, one has ∂*n*_eff_/∂*W* = 1.3 μm^−1^. For the TE_1_ mode, the value of ∂*n*_eff_/∂*W* drops from 1.2 μm^−1^ to 0.7 μm^−1^ when the core width increases from 0.7 μm to 1 μm. Even though one can reduce ∂*n*_eff_/∂*W* further by increasing the core width *W*, one should make a trade-off to avoid the reduction of the electro-optic bandwidth due to the increased capacitance in the resonator with broadened waveguides.

Figure 3(c) shows the calculated wavelength variation of a conventional MRM equipped with a 450-nm-wide bent waveguide and an FP-cavity modulator featuring a 1-μm-wide straight waveguide. Here, the random variation of the core width Δ*W* is assumed as ±0.5 nm and ±1 nm. In particular, the length *L*_wg_ is chosen as 0 μm and 30 μm, respectively, to give a comparison, showing that longer *L*_wg_ enables smaller wavelength variation, which is consistent with that discussed above. The reduction in resonant-wavelength shift compared with the MRM confirms the feasibility of embedding a broadened straight waveguide section in the FP-cavity modulator for minimizing the random wavelength variation. In contrast, it is inconvenient to introduce broadened waveguides for MRMs due to the difficulty of achieving sufficient coupling and depressing the higher-order mode excitation in the cavity. As it can be seen from Figure 3(c), the random resonant-wavelength variation for the FP-cavity with a broadened waveguide is much lower than MRMs with a single-mode waveguide in theory as desired.

### 2.4 PN junction

Owing to the introduction of the AMWGs to separate the input light and the output modulated light, both the TE_0_ mode and TE_1_ modes resonate in the FP cavity. Previously, a lateral PN junction is often employed in silicon modulators to facilitate overlap between the TE_0_ mode and the depletion region. However, selecting a lateral PN structure results in a minor overlap between the TE_1_ mode and the depletion region, consequently inducing a small phase shift for the TE_1_ mode. A vertical PN junction is preferable for both TE_0_ and TE_1_ mode modulation. However, the standard foundry does not provide the vertical PN junction due to its complex ion implantation process. Here, we introduce an interleaved PN junction combined with the lateral PN junction [42], as shown in Figure 1(d). This interleaved doping structure, characterized by periodic P- and N-doping with a PN period (Λ_pn_), differs from the conventional interleaved doping structure previously reported [45]. We introduce the parameter of *W*_e_ to achieve an additional lateral PN junction with an offset of *W*/2−*W*_e_. The additional lateral PN junction, featuring a PN offset, is advantageous for enhancing the per-length effective refractive-index variation Δ*n*_eff-TE1_ of the TE_1_ mode while preserving a high variation for the TE_0_ mode (Δ*n*_eff-TE0_). On the other hand, one should notice that the present interleaved doping structure might have a large parasitic capacitance when compared with lateral PN junctions, leading to bandwidth degradation, particularly for the structure with broadened waveguide. As a result, even though using the broadened waveguide helps reduce the random resonance-wavelength variation, one should make a trade-off by choosing the waveguide width appropriately to achieve improved resonance-wavelength uniformity and high bandwidth. Here, we choose the core width as 1 μm. The medium doping concentration was introduced at two sides of the rib waveguide with a distance of *W*_d_ from the edge of the rib waveguide. This distance is optimized to be 350 nm to reduce the sheet resistance of the PN junction and eliminate the carrier absorption loss induced by medium-concentrated doping. Another critical parameter of the interleaved doping is the doping period Λ_pn_, which is set to minimally 0.6 μm, aiming to enhance modulation efficiency for both modes, albeit at the expense of reduced bandwidth. It should be noticed that the bandwidth could be substantially compensated by the optical peaking enhancement effect [41]. The period for the interleaving doping is limited by the minimum 0.3 μm feature width for the P and N doping required by the foundry. Details about the simulation analysis of the modulation efficiency for the TE_0_ and TE_1_ modes with the varied parameters are given in Supplementary Material 4. Finally, the parameter *W*_e_ is set to 0.1 μm for a high modulation efficiency for TE_0_ and TE_1_ mode.

## 3 Experimental results

### 3.1 Static measurements

**Figure 4:**
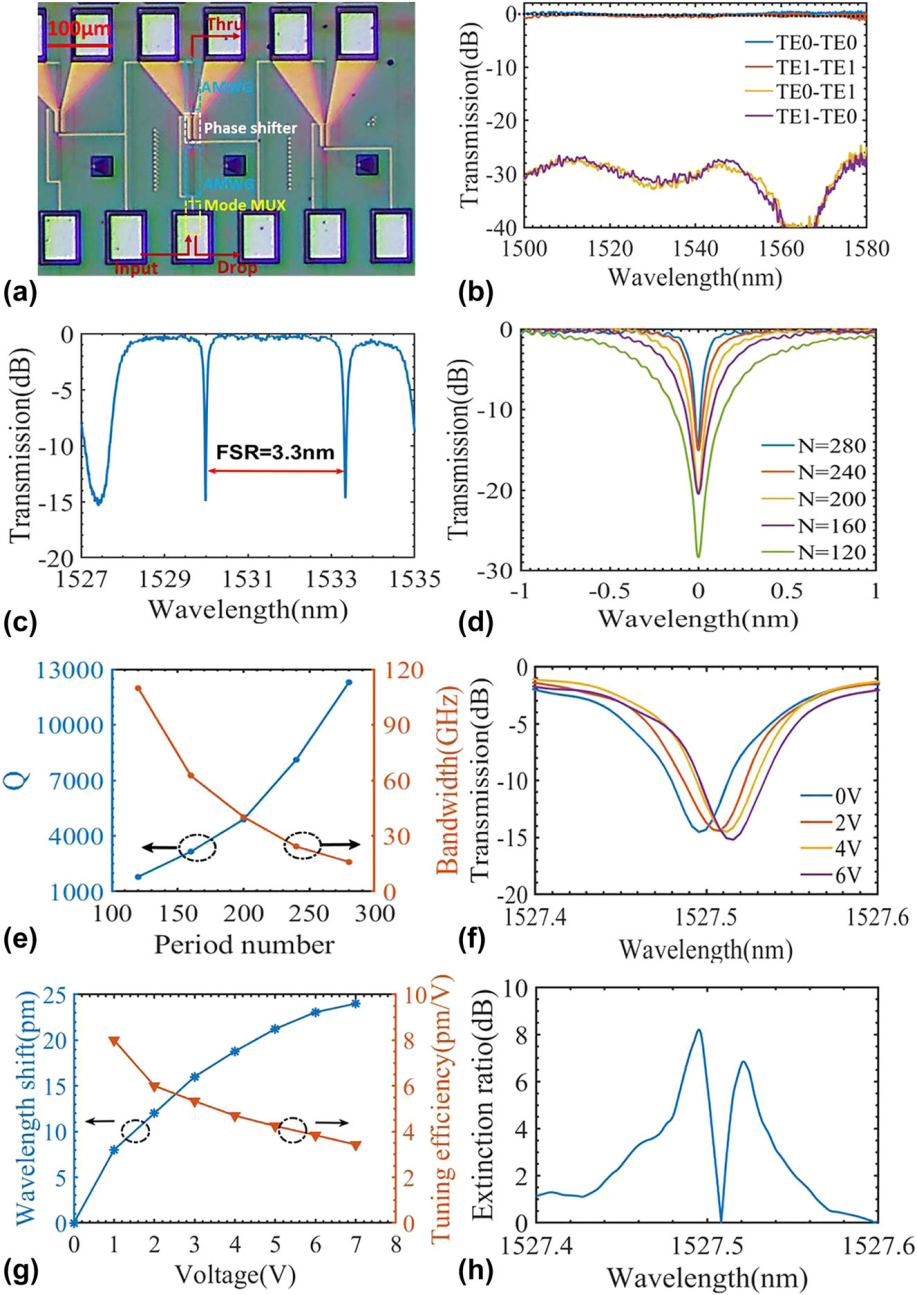
The measured results for the FP-cavity modulators. (a) Microscopic image of the fabricated FP-cavity modulators; (b) measured transmissions for mode (de)multiplexer. Inset: the test structure for mode (de)multiplexer; (c) measured spectral response at the drop port of the FP-cavity modulator; (d) measured spectral response near the resonance wavelength; (e) the extracted *Q* factor and *Q*-factor limited bandwidth for the different FP-cavity modulators with varied period numbers from 120 to 280; (f) measured normalized optical transmissions under different reverse bias voltages; (g) extracted wavelength shifts and the tuning efficiencies under different reverse bias voltages.

Figure 4(a) shows the microscopic image of the fabricated FP-cavity modulators. The input continuous light is launched from the input port and output from the drop port by a pair of grating couplers with a total of ∼10 dB coupling loss. The pads at the bottom of the devices function as the heating pad for tuning the centered Bragg wavelength of the AMWGs. The spectral responses were characterized utilizing an optical spectrum analyzer (OSA) and a broadband amplified spontaneous emission (ASE) light source. The transmission spectra of the devices were normalized by deducting the spectral responses obtained from a pair of grating couplers. Initially, the mode (de)multiplexer integrated within the FP-cavity modulator was characterized, as depicted in Figure 4(b). To assess the excess loss and crosstalk of the mode (de)multiplexer, two identically structured mode (de)multiplexers were interconnected, functioning as multiplexer and demultiplexer, respectively, as illustrated in the inset. The pair of mode (de)multiplexers exhibited a measured insertion loss of <1 dB and a crosstalk of <−25 dB. The spectral response at the FP-cavity modulator’s drop port (equivalent to the through port for MRM) was measured, as plotted in Figure 4(c). The FP-cavity modulator demonstrated an excess loss of less than 1 dB and a static extinction ratio of 15 dB. For the AMWG, increasing the period number from the designed value of 35 to the actual value of 280 becomes necessary for achieving sufficient reflectivity when fabricated with standard CMOS lithography. It is due to the optical proximity effects induced in the deep ultraviolet (DUV) lithography process, which tends to smooth the sharp corners of the designed Bragg grating structure and thereby reduce the equivalent corrugation depth. Consequently, the reflective bandwidth decreases from the designed value of 35 nm to the measured value of 7 nm and the FSR of the FP cavity decreases from 6.7 nm to 3.3 nm. Supplementary Material 5 discussed these discrepancies caused by the optical proximity effect when using the 193 nm DUV.

We also measured the spectral responses of the FP-cavity modulators with varied period numbers from 120 to 280. Figure 4(d) and (e) shows the measured transmissions near the resonance wavelength and the corresponding *Q* factors, respectively. It can be seen that the *Q* factor increases with the period number. When choosing the period number as 120, 160, 200, 240, and 280, the *Q* factor is about 1,800, 3,150, 4,900, 8,100, and 12,000, respectively, while the corresponding *Q*-factor limited bandwidths are 120 GHz, 62 GHz, 40 GHz, 24 GHz, and 16 GHz.

The measured finesse of the FP cavity is 25.6. The reduction in finesse compared with the simulated value results from the FSR of the fabricated device is as small as 3.3 nm and is beneficial for improving modulation efficiency. Additionally, the extinction ratio decreases as the period number increases. For example, as the period number increases from 120 to 280, the initially high extinction ratio of 28 dB declines to 15 dB, indicating that the FP cavity moves away from the critical coupling as the period number increases. In the present case, less period number is required for achieving the critical coupling regarding the absorption loss in the PN-doped section and the scattering losses due to the corrugation.

**Figure 5:**
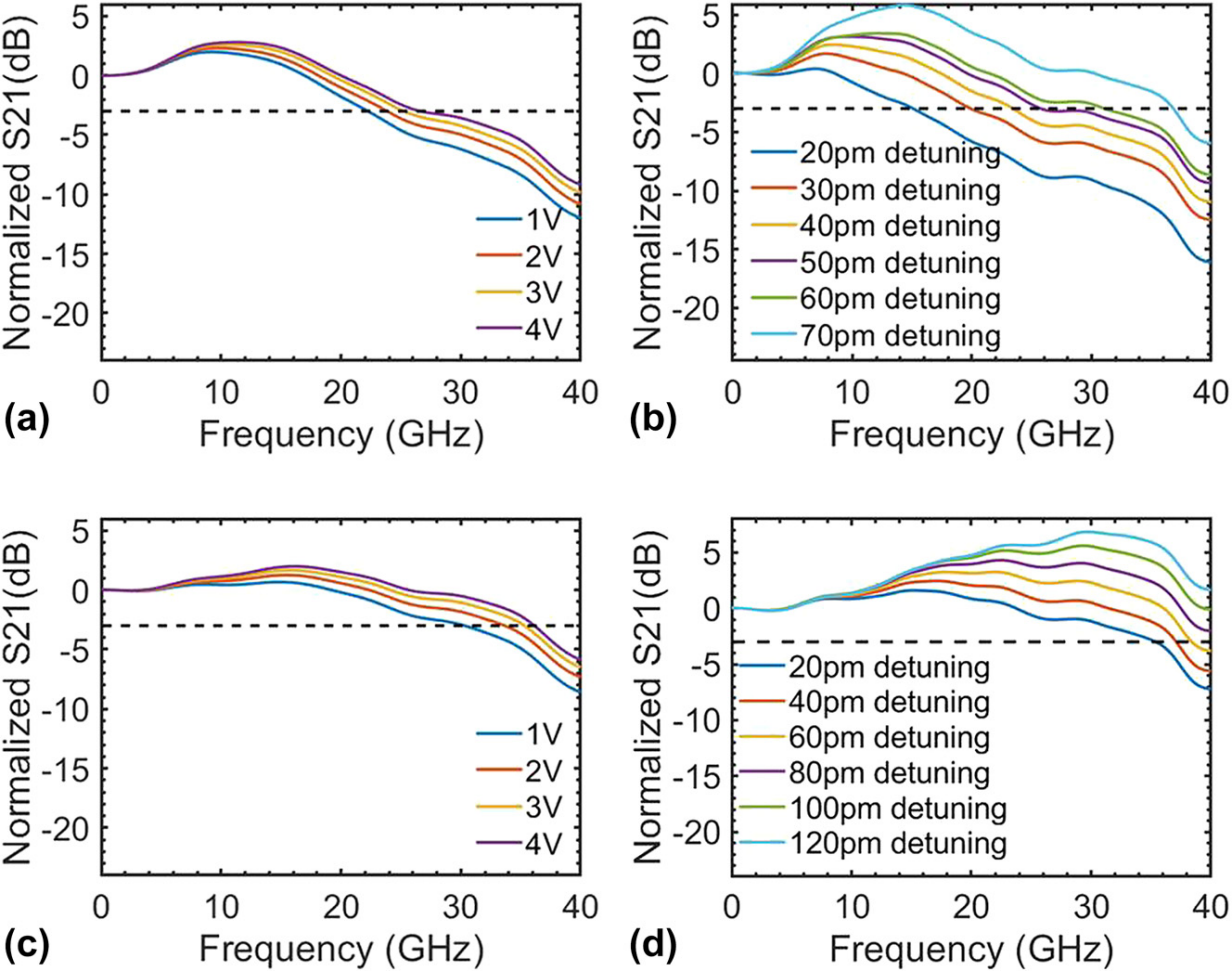
The bandwidth measurement results for the FP-cavity modulators. (a) The E-O bandwidth under different bias voltages at a fixed wavelength (40 pm wavelength detuning) for the FP-cavity modulator with period number of 280; (b) the E-O bandwidth under different wavelength detuning at a fixed 3 V bias voltage for FP-cavity modulator with period number of 280; (c) the E-O bandwidth under different bias voltages at a fixed wavelength (20 pm wavelength detuning) for FP-cavity modulator with period number of 240; (d) the E-O bandwidth under different wavelength detuning at a fixed 3 V bias voltage for FP-cavity modulator with period number of 240.

Here, the electric tuning efficiency for the FP-cavity modulator was also evaluated. Figure 4(f) shows the normalized optical transmission as the reverse bias voltage varies. The measured modulation efficiency is about 8 pm/V at 1 V, as shown in Figure 5(g). Therefore, the *V*_*π*_ *L* is determined to be 0.62 V cm under 1 V. The *V*_*π*_ *L* of the modulator increases with voltage, as the PN junction is gradually fully depleted under high voltage. For the present case, the effective cavity, which includes the segments of two AMWGs, extends beyond the central straight waveguide, suggesting that only a fraction of the cavity undergoes doping and is amenable to electrical tuning. Enhancement of the modulation efficiency is feasible by expanding the PN doping to include the AMWGs. Finally, considering the FP modulator may work with wavelength detuning from the resonance wavelength, we also extracted the measured dynamic extinction ratio versus the wavelength, shown in Figure 4(h). The extinction ratio reaches a maximum of 8 dB at the resonance wavelength and decreases with wavelength detuning.

### 3.2 High-speed measurements

Electro-optical (E-O) bandwidth and eye diagrams were measured to elucidate the high performance of the fabricated FP-cavity modulators. The E-O bandwidth under different bias voltages at a fixed wavelength and the E-O bandwidth under different wavelengths at a fixed bias voltage were measured for the FP modulator with a period number of 240 and 280. For the FP-cavity modulator with a period number of 280 (correspondingly, one has a moderate *Q* factor of 12,300 and a *Q*-limited bandwidth of 16 GHz), the bandwidth versus the voltage at a fixed wavelength with 40 pm detuning to the resonance wavelength was quantified and illustrated in Figure 5(a). The bandwidth increases from 22 GHz to 28 GHz as the voltage increases from 1 V to 3 V. The reduction in parasitic capacitance under a high reverse voltage, coupled with the increased wavelength detuning via electrical tuning, contributed to the improvement in bandwidth. When operated with increased voltages, the elevated S21 curves peaking at higher frequencies substantiate the optical peaking enhancement effect. Given the relatively low modulation efficiency of the FP-cavity modulator, the electrical tuning induced by the optical peaking enhancement effect in different voltages is poor. At higher voltages, the decrease in the parasitic capacitance markedly influences the enhancement of bandwidth.

Figure 5(b) shows the bandwidth versus different wavelength detuning when the reverse voltage is fixed at 3 V. In this experiment, the wavelength detuning ranged from 20 pm to 70 pm. The distinct optical peaking enhancement effect was corroborated in the proposed FP-cavity modulator, which was previously observed in MRMs [29], [41]. The *Q*-factor limited bandwidth is expected to be 16 GHz, as is shown in Figure 4(d), and the *RC*-limited bandwidth is calculated to be 17.5 GHz. The bandwidth without detuning is estimated to be 12.4 GHz under 3 V reverse bias, which is 3.4 GHz smaller than the bandwidth under 20 pm detuning. A more prominent optical peaking enhancement effect and larger bandwidth for the FP-cavity modulator are realized with larger wavelength detuning. With a wavelength detuning extended to 70 pm, the FP-cavity modulator achieves a bandwidth of 37 GHz accordingly. For the FP-cavity modulator with a period number of 240 (*Q* = 8,100, i.e., *Q*-limited bandwidth = 24 GHz), the bandwidth variations at different voltages and different wavelength detuning exhibit a pattern akin to that observed in the FP-cavity modulator with a period number of 280. E-O response to varied voltages was quantified when introducing a 20 pm wavelength detuning, as depicted in Figure 5(c). The bandwidth increases from 31 GHz to 36 GHz as the voltage increases from 1 V to 3 V. Figure 5(d) depicts the bandwidth measured at a fixed voltage of 3 V with a wavelength detuning varying from 20 pm to 120 pm. Upon selecting larger wavelength detuning, this FP-cavity modulator achieves a bandwidth exceeding 40 GHz, surpassing the capabilities of the Vector Network Analyzer (VNA) in the lab.

In summary, significant enhancement of bandwidth through the wavelength detuning underscores the potential for high-speed modulation, despite the FP-cavity modulator’s high *Q* factor and limited RC bandwidth. It is important to note that the bandwidth expansion achieved through wavelength detuning comes at the cost of a reduced extinction ratio, as shown in Figure 4(h). Greater wavelength detuning invariably leads to a diminished extinction ratio of the transmitter’s eye diagram. Consequently, a moderate wavelength detuning is often chosen for making a trade-off between the bandwidth and the modulation efficiency to realize open eye diagrams with a high extinction ratio.

**Figure 6:**
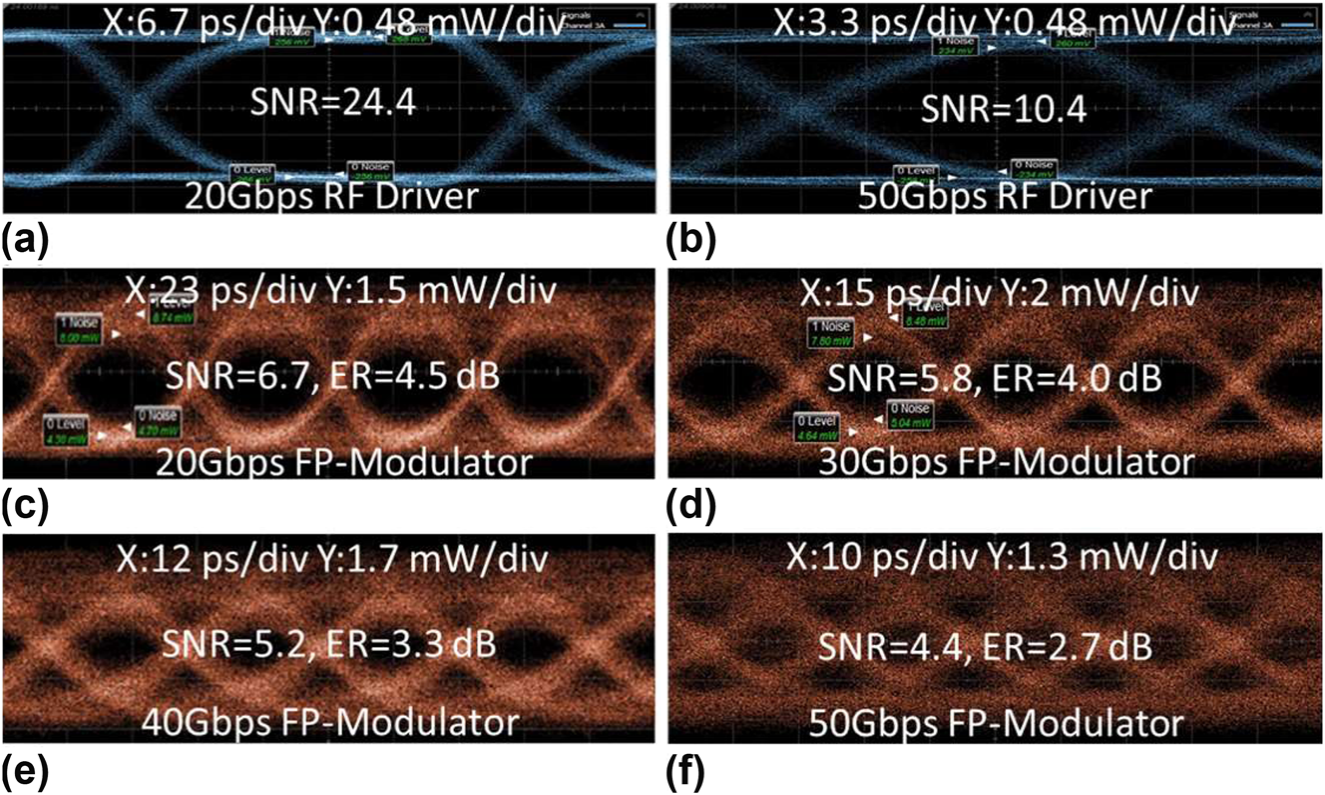
Measured NRZ eye diagram of the RF driver under (a) 20 Gbps and (b) 50 Gbps; measured NRZ eye diagram of the FP-cavity modulator with period number of 280 under (c) 20 Gbps, (d) 30 Gbps, (e) 40 Gbps, and (f) 50 Gbps.

Ultimately, high-speed eye diagrams of the fabricated FP-cavity modulator were obtained to corroborate its performance in high-speed data transmission. Eye diagrams were recorded at different bit rates varying from 20 Gbps to 50 Gbps, each under distinct wavelength detuning. Figure 6(a) and (b) shows the reference eye diagrams of the RF driver amplifier under 20 Gbps and 50 Gbps. Figure 6(c)–(f) shows the optical eye diagrams of the FP-cavity modulator at 20, 30, 40, and 50 Gbps, respectively. The eye diagrams were measured for the FP-cavity modulator with a period number of 280, which has a low bandwidth and requires wavelength detuning to support high-speed modulation. Wavelength detuning increased with higher bit rates to enhance the bandwidth, albeit at the expense of reduced extinction ratio. Improved eye diagrams could be anticipated by further extending the PN junction to encompass the two AMWG sections, thereby improving the modulation efficiency; concurrently, reducing the period number may allow higher bandwidth and preserve an acceptable modulation extinction ratio.

### 3.3 Wafer-level measurements

**Figure 7:**
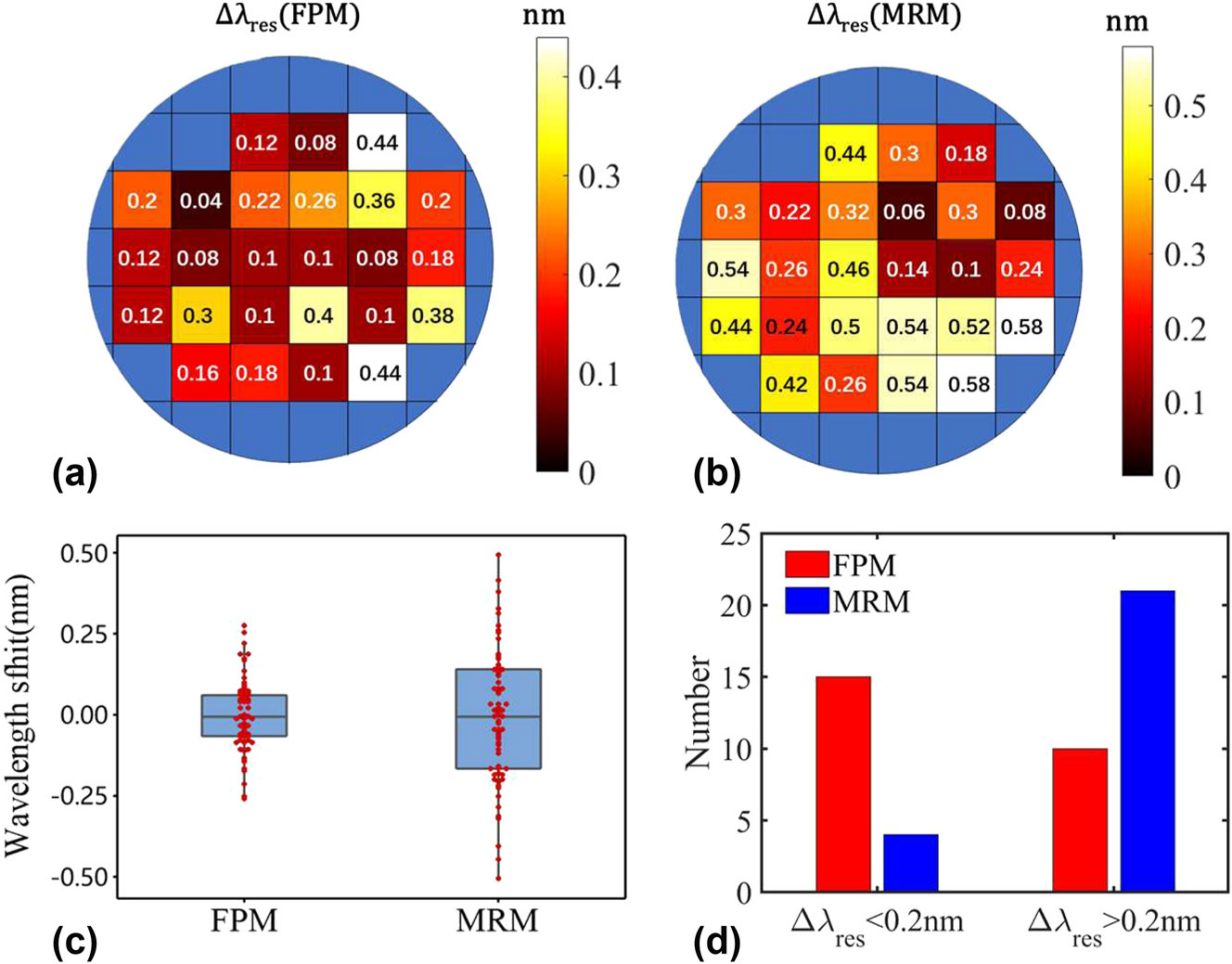
The wafer-level measurement of the resonance-wavelength variation (a) for FPMs (Δ*λ*(FPM)) and (b) for MRMs (Δ*λ*(MRM)) on each chip; (c) the box plot of the resonance-wavelength variation for FPMs and MRMs across 25 chips; (d) the number of chips on which the resonance-wavelength variation is larger than 0.2 nm and less than 0.2 nm for FPMs and MRMs, respectively.

An 8-inch wafer-level measurement encompassing 25 chips was performed to record the resonance wavelengths of both MRMs and FPMs on the same chips. Each chip contained three identical MRMs and FPMs, spaced 200 μm between adjacent devices. The maximum resonance-wavelength variation Δ*λ*_res_ of the fabricated FPMs and MRMs on the same chip was recorded, and the corresponding wafer maps are, respectively, shown in Figure 7(a) and (b). According to the foundry’s datasheet, the MRMs equipped with 0.45-μm-wide waveguides exhibit an average wavelength variation of 0.55 nm. In contrast, the resonance-wavelength variation can be reduced to Δ*λ*_res_ = 0.34 nm by using the MRMs developed here with a waveguide whose core width is widened to 0.65 μm, as shown in Figure 7(b). As a comparison, the wafer-level average resonance-wavelength variation is further reduced by 43 % to Δ*λ*_res_ = 0.19 nm when using the present FPMs. Figure 7(c) shows the box plot of the resonance-wavelength variation for FPMs and MRMs across 25 chips. Evidently, the FPMs achieve a significant reduction in random wavelength variation compared to the MRMs. We also count the chips exhibiting a resonance-wavelength variation greater than 0.2 nm and less than 0.2 nm for FPMs and MRMs, respectively, as depicted in Figure 7(d). For FPMs, there are 15 out of 25 chips demonstrated a minor variation of less than 0.2 nm. In contrast, for MRMs, there are only 4 out of 25 chips feature a variation of less than 0.2 nm. In other words, 60 % of the FPMs feature Δ*λ*_res_ < 0.2 nm, whereas only 16 % of the MRMs feature Δ*λ*_res_ < 0.2 nm. To conclude, the wafer-level measurement verified the advancement of the FPMs developed here in reducing resonant-wavelength variation. This 0.15 nm resonance wavelength variation reduction can reduce the power consumption of the heating tuning for wavelength alignment, as the heating for wavelength alignment accounts for most of the power consumption in the resonant modulator. Considering the 0.11 nm/mW heating tuning efficiency and 50 Gbps transmitting, the FPM shows an overall estimated 22 fJ/bit power consumption reduction compared with the MRM. The detailed power consumption discussion for the FPM and MRM can be referred to Supplementary Material 3.

## 4 Discussions

In comparison to traditional 1 × 1 FP cavities [37], the present 2 × 1 FP-cavity modulator exhibits significant scalability, enabling the extension in cascade for implementing a DWDM transmitter with multiple wavelength channels [46], as these modulators can be cascaded to modulate and multiplex multiple data channels simultaneously, while also reducing the energy consumption required for wavelength alignment. Furthermore, the anticipated larger FSR of the FP-cavity modulator holds promise for supporting a greater number of WDM channels compared to conventional MRMs. The inherent advantages of the FP cavity structure also offer competitive benefits when used to work as resonant all-silicon photodetectors. The concept of an FP-cavity-based WDM transceiver, featuring a larger FSR and lowered fabrication sensitivity, presents a viable alternative to conventional MRM-based transceivers [47]. Furthermore, the proposed FP cavity can be adapted for use in wavelength-selective optical switches and reconfigurable optical add-drop multiplexers (ROADM) [48]. In such a case, a more broadened waveguide can be chosen to further improve the fabrication tolerance with a reduced random resonance-wavelength variation. Besides, compared with the previously reported conventional 1 × 1 FP cavity [49], [50], the present 2 × 1 FP cavity obviates the need for an additional optical circulator, promising enhanced feasibility for monolithic integration of optical transmitters.

The critical aspect of the FP-cavity modulator centers on the fabrication of the AMWGs, which necessitates finely teeth within the waveguide. Primarily, open-access MPW foundries support only 130 nm process nodes, posing challenges in fully fabricating these delicate structures. The associated optical proximity effect, which critically influences the coupling coefficient of the AMWG, results in a discrepancy between the designed and fabricated devices. We assert that employing an advanced fabrication process with smaller feature sizes or utilizing a Bragg grating with greater corrugation depth would further enhance the performance of the FP-cavity modulator. In such scenarios, the coupling coefficient of the AMWG would be enhanced, thereby reducing the number of periods required to achieve the AMWG’s desired reflectivity. Consequently, this allows for a substantial reduction in the cavity length, achieving an increased FSR that even exceeds that of MRMs. Besides, if advanced vertical PN doping is accessible, it would facilitate a reduction in the parasitic capacitance typically induced by interleaved doping beneath a wide waveguide and improve the modulation efficiency. Additionally, the modulation efficiency of the FP-cavity modulator could be enhanced by extending the PN junction into the AMWGs, thereby encompassing a large portion of the cavity for effective modulation.

Table 1 summarizes the reported silicon photonics modulators with compact footprints. The slow-light, folded-waveguide, and resonant structures have successfully minimized the length of the phase shifter region to a lumped dimension. Among them, the slow-light modulator suffers from a high insertion loss, while the folded-waveguide modulator is constrained by the low modulation bandwidth. In contrast, the resonant modulator has the advantages of high modulation bandwidths and ultra-compact footprint. Conventionally, the MRMs have been extensively developed over past decades, while FP-cavity modulators should be considered as an attractive alternative to date. Due to the waveguide bends indispensable in MRMs, one has to choose a relatively long cavity and relatively narrow waveguides. As a result, MRMs often have quite limited FSRs and relatively large random wavelength variations. For FPMs with a straight cavity, the length can be shortened to extend the FSR and a broadened waveguide beyond the single-mode regime is allowed for alleviating the sensitivity to the variations of waveguide dimensions. Experimentally, the presented FPM boasts a compact of 30 μm for active region, which is one-half of the MRMs. Considering the long length of the AMWGs and mode (de)multiplexer, the total length of the FPM increases to 250 μm. Nevertheless, the calculated area of FPM is still as small as 0.0015 mm^2^, which is comparable to that of MRMs as well. Furthermore, the wafer-level measurements also indicate that the FPMs exhibit a 43 % improvement in performance concerning random resonance-wavelength variation when compared to the MRMs.

**Table 1:** Summary of the reported compact silicon photonics modulator.

Reference	Type	Length^a^ (μm)	Area (mm^2^)	Bandwidth (GHz)	*V*_*π*_ *L* (V cm)	Insertion loss (dB)
[19]	Slow-light	/, 200	/	31	/	5
[20]	Slow-light	350, 50	0.225	/	/	9.1
[21]	Slow-light	/, 500	/	/	0.85	6
[22]	Slow-light	/, 570	/	42	0.51	5.5
[23]	Folded-waveguide	490, 1,100	0.07	26	2.1	4
[24]	Folded-waveguide	700, 250	0.28	17.3	0.85	3.7
[25]	Folded-waveguide	650, 250	0.29	31	0.93	1.22
[26]	MRM	150, 250	0.0075	19	0.76	0.2
[27]	MRM	/, 94	0.001	11	1.5	2
[28]	MRM	/, 47	0.0004	16.3	/	/
[29]	MRM	/, 60	/	50^b^	0.825	/
[30]	MRM	/, 62.8	/	50^c^	0.52	/
[49]	1 × 1 FPM	/	/	1.3	/	1
[50]	1 × 1 FPM	1,580, 1,240	0.205	5.6	/	9.4
**This work**	**1 × 2 FPM**	**250, 30**	**0.0015**	**>40** ^**d**^	**0.62**	**<1**

^a^The first number is the total length of the modulator, the second number is the length of the active phase shifter region; ^b^the bandwidth was tested under 0.115 nm wavelength detuning; ^c^the bandwidth was tested under unknown wavelength detuning; ^d^the bandwidth was tested under 0.08 nm wavelength detuning.

## 5 Conclusions

In this paper, a novel FP-cavity silicon photonic modulator with a 30-μm-long compact size and 1-μm-wide broadened waveguide was proposed and realized to demonstrate its advantage of less random resonance-wavelength variation. The proposed device was successfully validated in a standard silicon photonics foundry. By introducing two AMWGs as reflectors within the FP cavity to enable additional TE_0_–TE_1_ mode conversion between the input and reflective light, alongside an adiabatic mode (de)multiplexer to separate the output modulated and input light, a 2 × 1 FP-cavity was achieved with no extra optical circulator required. An interleaved doping structure was implemented to facilitate high modulation efficiency for both TE0 and TE1 modes. The proposed device shows a measured insertion loss of less than 1 dB and an extinction ratio of 15 dB. The modulation efficiency was measured to be 0.62 V cm under −1 V, comparable to that of the conventional MRMs. The bandwidth of the FP-cavity modulators under different wavelength detuning was also measured to demonstrate the optical peaking enhancement effect observed in the previously reported MRMs. The measurement results show a bandwidth of 28 GHz and 36 GHz for the FP-cavity modulator with periods of 240 and 280, respectively. By further increasing the wavelength detuning, a bandwidth of over 40 GHz is observed at the cost of lowering modulation efficiency. Eye diagrams at 20, 30, 40, and 50 Gbps were measured to demonstrate the high-speed modulation capabilities. Finally, wafer-level measurements were conducted for both MRMs and FPMs, revealing that FPMs have an average improvement of 43 % in random resonance-wavelength variation than MRMs. In summary, the proposed ultra-compact silicon photonics FP-cavity modulators exhibit enhanced performance in reducing resonance-wavelength variation, which is useful for future applications.

## Supplementary Material

Supplementary Material Details
