# Supplementary material for: Silicon photonic modulators with a 2 × 1 Fabry–Perot cavity
Source: Nanophotonics. 2025 Jan 14;14(2):183–96. doi: 10.1515/nanoph-2024-0488 (PMC11806507; doi:10.1515/nanoph-2024-0488)
Supplement: Supplementary file 1 — Supplementary Material Details [file j_nanoph-2024-0488_suppl_001.docx]

Silicon Photonic modulators with a 2×1 Fabry-Perot cavity: Supplemental material

Hengzhen Cao^1^, Jin Xie^1^, Weichao Sun^1^, Mingyu Zhu^1^, Yuluan Xiang^1^, Gong Zhang^1^, Jingshu Guo^1,2^, Yaocheng Shi^1,2^, and Daoxin Dai ^1,2,3, *^

*^1^ State Key Laboratory of Extreme Photonics and Instrumentation, College of Optical Science and Engineering, International Research Center for Advanced Photonics, Zhejiang University, Zijingang Campus, Hangzhou 310058, China*

*^2^ Jiaxing Key Laboratory of Photonic Sensing & Intelligent Imaging, Intelligent Optics &Photonics Research Center, Jiaxing Research Institute, Zhejiang University, Jiaxing 314000, China*

*^3^ Ningbo Research Institute, Zhejiang University, Ningbo 315100, China.*

*^*^*[*dxdai@zju.edu.cn*](mailto:dxdai@zju.edu.cn)

1. Mode (de)multiplexer design

The mode (de)multiplexer we selected is an adiabatic dual-core tapered coupler for large fabrication tolerance and wavelength-insensitive operation [1]. Fig.S1(a) shows the schematic configuration of the mode (de)multiplexer. The structure comprises a wide core waveguide (waveguide A) and a narrow coupling waveguide (waveguide B). The mode (de)multiplexer principle is based on the adiabatic evolution of supermodes. When the TE_0_ mode is launched at the right side of waveguide A, it will straightly go through the adiabatic tapered region without coupling to waveguide B. The TE_1_ mode will be gradually coupled to waveguide B and adiabatically converted to TE0 mode with no excess loss. The critical parameters of the mode (de)multiplexer are the widths of the waveguide A and B in the adiabatic coupling region. From the supermodes theory perspective, the core widths at the right side of waveguide A and B are chosen as *w*_a2_>>*w*_b2_, in which case the supermodes are mainly localized in waveguide A. The core widths at the left side of waveguides A and B are chosen as *w*_a1_>*w*_b1_ so that the supermodes generated at the right side would evolve adiabatically and transfer to the corresponding supermodes at the left side, which are mainly localized in the waveguide B. The gap (W_g2_) between the waveguides A and B in the coupling region should be small enough for easy coupling between the two waveguides. The length of the coupling region should be long enough for adiabatic evolution. Besides, considering the sidewall angle of the waveguide fabricated by the foundry is approximately 87.5°, the mode hybridness between TE_1_ and TM_0_ would excite if the width of waveguide A is appropriate. Fig.S1(b) shows the calculated results of the dispersion curves when the waveguide was operated at 1550 nm. The dashed circle in Figure.S1(b) indicates the mode hyridness region where TE1 mode would convert to TM mode in the long-tapered waveguide. In our design, the widths of the two waveguides are set to (*w*_a1_, *w*_b1_) = (0.42, 0.28) μm and (*w*_a2_, *w*_b2_) = (0.6, 0.12) μm. The gap widths *w*_g1_, *w*_g2_, and *w*_g3_ are chosen as 1.5, 0.18, and 1.8 μm, respectively, while the taper lengths *L_01_*, *L_12_*, and *L_23_* are chosen as 20, 35, and 15 μm, respectively. The gap width at the adiabatic coupling region is set to 0.18 μm to prevent incomplete etching and ensure the cladding can easily fill the gap in the standard fabrication process. Fig.S1(c)(d) shows the simulated light propagation in the designed adiabatic dual-core taper by three-dimensional finite-difference time-domain (3D-FDTD) when the TE_0_ and TE_1_ modes are launched at the right side of the waveguide A respectively. The corresponding simulated transmissions at the left side of the mode (de)multiplexer are also shown in Figure.S1(e). The designed mode (de)multiplexer has a low excess loss of less than 0.1 dB and a low crosstalk of <-30 dB in the wavelength band from 1500 to 1600 nm.


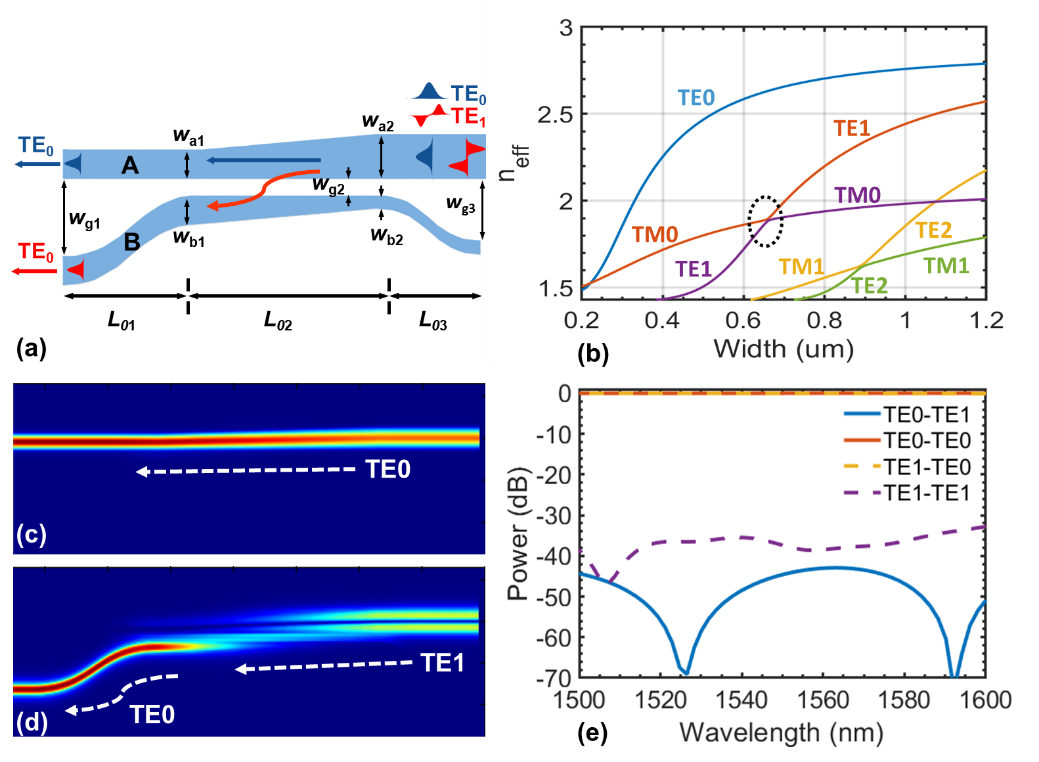


**Fig.S1.** (a) Schematic of the selected mode (de)multiplexer;(b) Dispersion curves of the strip waveguide under 1550nm for different modes. The dashed circle indicates the mode hybridness region; Simulated light propagation in the designed mode (de)multiplexer when the (c) TE_0_ mode and (d) TE_1_ mode are launched at the right side of the waveguide A;(e) The corresponding simulated transmissions at the left side of waveguide the mode (de)multiplexer.

**2. The details for extracting the Q factor of the FP cavity**

The Q-factor of the FP-cavity modulator is derived by fitting the measured reflection spectrum with the following equation:

$\left| \frac{E_{out}}{E_{in}} \right|^{2}=\left| 1-\frac{\mu^{2}}{-i\left( \omega_{0}-\omega_{r} \right)+\frac{1}{\tau_{a}}} \right|^{2}$  (S1)

Where *E*_in_ and *E*_out_ are the field amplitude of the input and reflected light, *μ* is the coupling coefficient between the input waveguide and the FP cavity, *ω*_o_ and *ω*_r_ refer to the light frequency and the cavity resonant frequency, and *τ*_a_ is the 1/e^2^ decay time of the cavity field intensity (here *τ*_a_ = 2*Q*/*ω*_r_).

Once the simulated Q factor of the FP cavity is obtained, we can calculate the corresponding Q-factor-limited bandwidth in Figure 2(d) using the following equation.

$f_{Q}=\frac{c}{\omega*Q}$ (S2)

3. Parasitic capacitance and power consumption estimation

We selected a novel interleaved doping distribution that simultaneously maintains a high modulation efficiency for TE_0_ and TE_1_ modes [2]. It should be noted that interleaved doping features a much larger parasitic capacitance compared with conventional lateral doping. Also, the FP modulator we present has a wider waveguide of 1 μm width to demonstrate the advantages of the less resonance wavelength shift. The wider waveguide and interleaved doping distribution result in a larger parasitic capacitance per length. Thus, the RC-limited bandwidth could not be ignored for total bandwidth estimation. A comprehensive calculation of the parasitic parameters is necessitated to evaluate the bandwidth. Generally, the PN junction capacitance can be regarded as a plate capacitor (denoted as *C_||_*) whose width of the capacitor is determined by the depletion width. However, for the 220nm-thick SOI platform, the fringing electric field caused by the wider depletion regions near the top and bottom surfaces of the silicon waveguide would result in two equivalent fringe capacitances (denoted as *C_cpst_* and *C_cpsd_*). The fringe capacitance due to the charges at the center of the PN junction should also be considered (denoted as *C_f_*) [3]. Fig.S2(a) shows the calculated parasitic capacitance per length under different doping concentrations for 1 μm wide waveguide. The lateral doping and interleaved doping with different doping concentration periods were all calculated for varying the doping concentrations (1e17 cm^-3^ to 1e19 cm^-3^), as shown in Fig.S2(b). The capacitance increases with the smaller PN period. When the PN period reaches 0.6 μm, the interleaved PN junction has a high capacitance per length of 0.54 fF/μm and 1.15 fF/μm for light doping (3e17 cm^-3^) and middle doping (2e18 cm^-3^) concentration situation under -3 V. In contrast, the lateral PN junction only has a capacitance of 0.2 fF/μm and 0.4 fF/μm accordingly. The interleaved doping exhibits 2.5 times larger capacitance than lateral doping, indicating a reduction in RC-limited bandwidth. The parasitic resistance can be regarded as two sheet resistances in the slab of the rib waveguide (denoted as *R*_p_ and *R*_n_). Thus, we could evaluate the RC-limited bandwidth using the equation *f*=1/2π(*R_p_*+*R_n_*)*C*, where *C*=*C_||_*+*C_f_*+*C_cpst_*+*C_cpsd_*. For the 1 μm wide waveguide and 0.6 μm doping period, the proposed FP modulator has a calculated low RC-limited bandwidth of 19.7 GHz and 9.1 GHz for light and medium doping concentration under -3 V reverse voltage, respectively. Though the medium doping concentration performs better in modulation efficiency, we still choose the light doping concentration to balance the bandwidth and modulation efficiency. Also, the cavity's intrinsic loss under middle doping concentration is too large to achieve critical coupling when the FP modulator works at a moderate *Q*-factor (i.e., *Q*=4000~10000). For the FP modulator with a 30 μm-long phase shifter region, the calculated parasitic capacitance under the low doping concentration we selected is 16.2 fF, and the corresponding dynamic power consumption under 2.1V peak-to-peak voltage is 17.9 fJ/bit. The large parasitic capacitance per length introduced by interleaved doping is responsible for the large power consumption. In contrast, the dynamic power consumption of the micro-ring modulator with a bending radius of 10 μm is 13.2 fJ/bit. Though the proposed FP modulator features a larger electric-tuning consumption compared to the typical micro-ring modulator, it still should be noted that the heat-tuning power consumption for wavelength alignment mainly influences the total power consumption of the resonant type modulator. The FP modulator features an average 0.15 nm wavelength variation reduction compared with the micro-ring modulator. For the 50 Gbps transmitting, the corresponding heating power consumption reduction is estimated to be 27 fJ/bit. Considering the FP modulator exhibits 17.9-13.2=4.7 fJ/bit more electric tuning power consumption compared with the conventional microring modulator, the FP modulator shows a great 27-4.7=22.3 fJ/bit power consumption reduction. It can be seen the less random wavelength shift by the FP modulator can significantly reduce the heat-tuning power consumption and thus improve the resonant modulator's total power consumption.


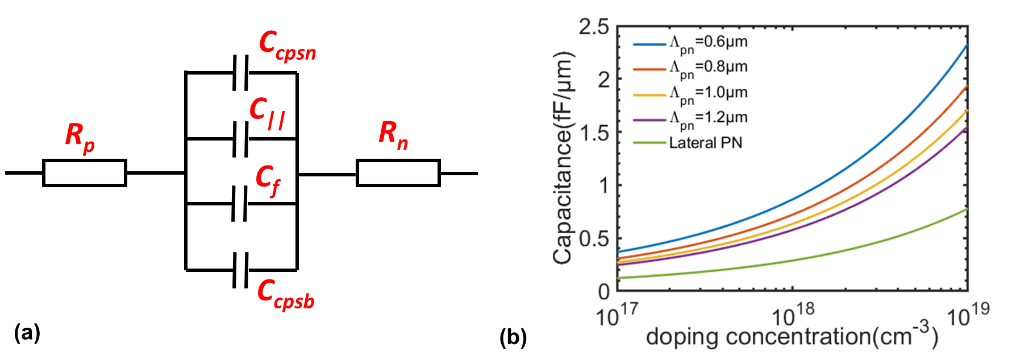


**Fig.S2.** (a)The equivalent parasitic model for the interleaved PN junction; (b) Simulated parasitic capacitance per length for the interleaved doping with different PN periods and the lateral doping under different doping concentrations.

4. Electro-optic modulation for the interleaved doping

To evaluate the electro-optic modulation efficiency for the presented interleaved doping, the effective refractive index variations versus the varied PN period(*Λ*_pn_) and *W*_e_ for both TE_0_ and TE_1_ modes were also simulated. Fig.S3(a)(b) shows the simulated *Δn*_eff_(TE_0_) and *Δn*_eff_(TE_1_) versus PN period. Both *Δn*_eff_(TE_0_) and *Δn*_eff_(TE_1_) increase with a smaller PN period. The PN period has a greater influence on Δn_eff_(TE_0_). When the PN period is as small as 0.6 μm, *Δn*_eff_(TE_0_)=1.6e-4 and *Δn*_eff_(TE_1_)=2e-4, which shows a 60%(i.e., *Δn*_eff_(TE_0_)=1e-4 for lateral PN junction) and 440% (i.e., *Δn*_eff_(TE_1_)=4.5e-5 for lateral PN junction) improvement for TE_0_ and TE_1_ mode respectively. The greater improvement for *Δn*_eff_(TE_0_) mode compared to the previously reported interleaved doping is attributed to the introduction of the lateral offset PN junction by parameter *W_e_*, as well as the wider waveguide that results in a smaller *Δn*_eff_(TE_0_) compared to the normal wide waveguide for lateral PN. The larger depletion area overlapping with the TE_1_ mode optical field distribution accounts for the huge improvement for *Δn*_eff_(TE_1_). The influence of the lateral PN offset (*W*_e_) was also investigated and simulated, as shown in Fig.S3(c)(d) for TE_0_ and TE_1_ mode. The simulation shows that when *W*_e_=0.1 μm, the TE_0_ and TE_1_ modes would have a higher *Δn*_eff_ than the conventional interleaved doping. When W_e_ is further increased, *Δn*_eff_(TE_1_) decreases greatly under high reverse voltage, and *Δn*_eff_(TE_0_) increases slightly.


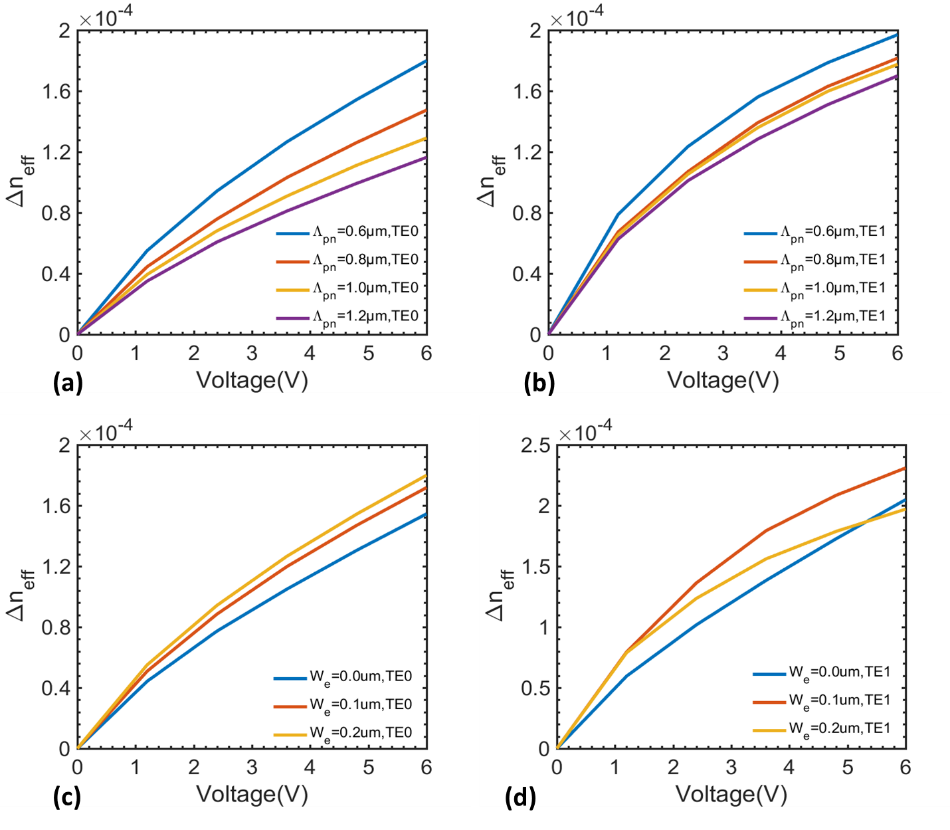


**Fig.S3.** Simulated effective refractive index variation for the interleaved doping with different PN periods(*Λ*_pn_) for (a) TE_0_ mode and (b) TE_1_ mode; Simulated effective refractive index variation for the interleaved doping with different lateral PN offsets (*W*_e_) for (c) TE_0_ mode and (d) TE_1_ mode.

**5. The discussion of the large discrepancy between the designed and measured results**

We spotted that the actual reflection bandwidth is reduced to approximately 7 nm, only one-fifth of the designed value of 35 nm. The measured FSR is only 3.3 nm, only one-half of the designed value of 6.7 nm. In addition, the FP cavity under test has a corrected period number of 280, which is 8 times larger than the designed value of ~35. Fig.S4 shows the simulated and measured spectrum response for the proposed FP cavity modulator to demonstrate this discrepancy. The centered wavelength difference between the simulated and measured results caused by the silicon thickness is ignored and normalized.


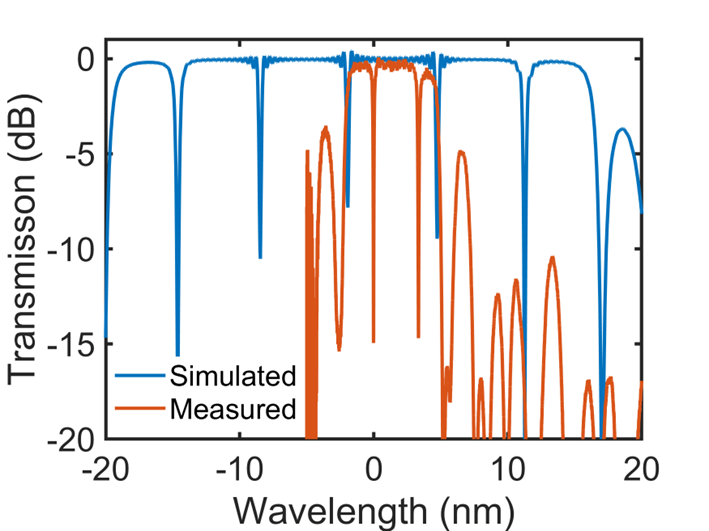


Fig.S4 Simulated and measured spectrum response for the proposed FP cavity modulator.

The correction for period number is necessitated when fabricating devices using standard CMOS lithography instead of electron beam lithography (EBL). This is due to the optical proximity effects generated by 193 nm Deep Ultraviolet Lithography (DUV), which would smooth the sharp corners of the designed Bragg grating structure and reduce the equivalent corrugation depth. As a result, the coupling coefficient(κ) of the MWG decreases significantly. The period number should increase following the decrease of *κ* to maintain the same reflectivity$(R={tanh}^{2} \left( \kappa L \right))$ and achieve a moderate *Q*-factor. According to the coupled-mode theory, the Bragg reflection bandwidth is influenced by the coupling coefficient(*κ*) and the grating length(*L*), as described below [4]:

$\Delta\lambda=\frac{\lambda_{0}^{2}}{\pi n_{g}L}\sqrt{\kappa^{2}L^{2}+\pi^{2}}$ (S3)

Where *n_g_* is the group refractive index. When the same $\kappa L$ is required to achieve the same reflectivity, the optical bandwidth is influenced by the grating length (i.e., period number). Consequently, the larger period number we selected to compensate for the optical proximity effects results in the reflection bandwidth reduction. The weaker coupling coefficient, which results in more period numbers needed to achieve the desired reflectivity with the corresponding longer cavity, is also responsible for the reduced measured FSR compared to the designed value.

Here, we also give a discussion about eliminating this great discrepancy between the designed and measured results. A triangular grating profile [5] can be introduced to reduce the optical proximity effects and eliminate the large discrepancy between the designed and fabricated devices. Nevertheless, a larger corrugation depth and more period number are needed to compensate for the corresponding smaller coupling coefficient. Similarly, a rectangular grating profile with larger corrugation depth may also benefit the devices with larger FSR and optical bandwidth [6]. Besides, advanced computational lithography for silicon photonics design is preferable to compensate for optical proximity effects [7].

6. Equivalent circuit of the FP modulator

The equivalent circuit of the FP modulator was also analyzed and extracted by measuring the S11 curve of the FP modulator. The VNA measured the S11 curve and the open-short-load calibration was conducted using the calibration plate provided by the RF probe's vendor. Fig.S5(a) shows the equivalent circuit of the FP modulator, which is similar to the micro ring modulator [8]. In this circuit model, *C_j_* is the capacitance of the PN junction, and *R_s_* is the series resistance. *C_ox_* is the buried oxide capacitance, and *R_si_* is the substrate resistance. The parasitic inductance, resistance, and capacitance of the metal pad and vias are denoted as *L_p_*, *R_p,_* and *C_p_*, respectively. The S11 curve was measured under 4 V reverse voltage, as shown in Fig.S5(b). The fitted curve is also shown in Fig.S5(b) as well. The corresponding extracted parameter of the equivalent circuit is displayed in Table S1. The extracted value for the junction capacitance (*C_j_*) and the series resistance (*R_s_*) is consistent with our simulated value. The series resistance is much larger than the typical micro ring modulator and is a consequence of the shorter active phase shifter and the larger resistivity for the light doping concentration used in the FP modulator. The shorter active phase shifter and the light doping for the interleaved PN junction also produce low junction capacitance with low power consumption.

Table S1.The extracted parameter of the FP modulator.

| *L_p_* | *R_p_* | *C_p_* | *R_s_* | *C_j_* | *R_si_* | *C_ox_* |
| --- | --- | --- | --- | --- | --- | --- |
| 10 pH | 14.2 Ω | 29 fF | 451 Ω | 17.4 fF | 1700 Ω | 20 fF |


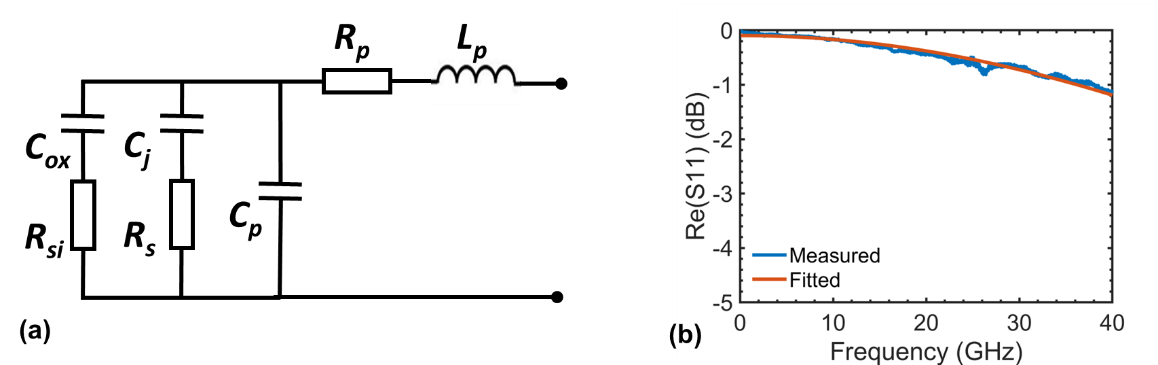


**Fig.S5.** (a)The equivalent circuit model of the FP modulator; (b)The measured and fitted real part of the S11 curve for the FP modulator under 4 V reverse voltage.

7. High-speed experimental setup

The experimental setups for the bandwidth and eye-diagram measurement are shown in Fig.S(a)(b), respectively. For the bandwidth measurement shown in Fig.S(a), the vector network analyzer (VNA, ROHDE&SCHWARZ ZVA40) combined with a commercial high-speed photodetector (PD, albis PQS40A-L) was employed to get the S21 curves of the modulator. The influence of the cables was excluded by SOLT calibration before test, and the S21 curve of the commercial high-speed photodetector was subtracted. For the high-speed eye-diagram measurement shown in Fig.S(b), an OOK-type pseudo-random bit sequence (PRBS) with Non-Return-Zero (NRZ) format is generated by a high-speed arbitrary wave generator (AWG, Micram DAC10002). The output signal with 400 mV peak-to-peak voltage is amplified by a high-speed RF amplifier (SHF S807C). A source meter (Keithley 2400) provides the reverse-biased voltage for the FP modulator. The amplified RF signal and the bias voltage are connected to a bias-T (Anritsu K251) whose output port is connected to the FP modulator via a GS-type RF probe. Considering the output optical power is relatively low for detection, the erbium-doped fiber amplifier (EDFA, KEOPSYS CEFA-C-BD) and narrow-band optical filter are used to amplify the optical signal and reduce the noise induced by the EDFA, respectively. The amplified modulated optical signal is detected by the optical port of the sampling Oscilloscope (Agilent 86100D).


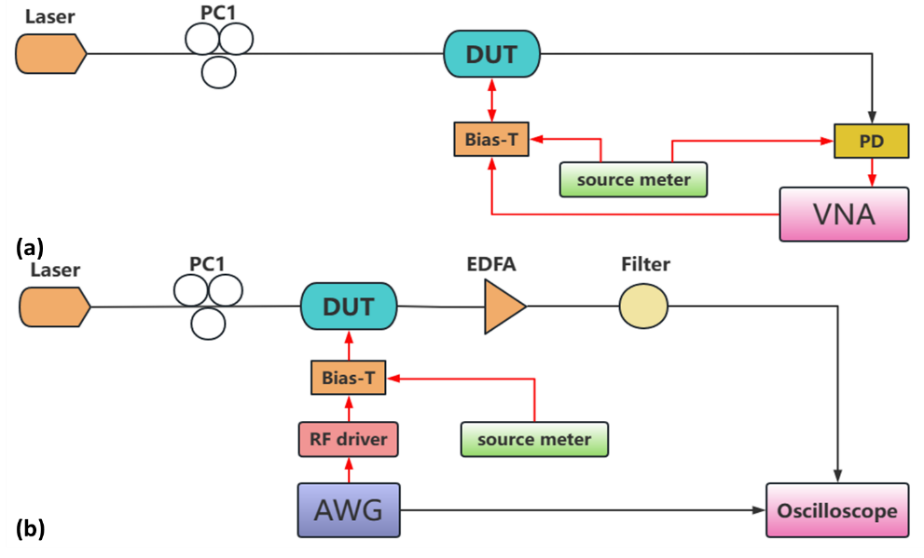


**Fig.S6.** (a) Experimental setup for E-O bandwidth measurement. Inset: the microscopic image of the fabricated FP modulators under test;(b) Experimental setup for eye-diagram measurement. The black and red lines represent the optical and electrical connections, respectively. DUT, device under test; AWG, arbitrary waveform generator; EDFA, erbium-doped fiber amplifier; VNA, vector network analyzer; PC, polarization controller; PD, photodetector.

8. Wafer-level measurement for centered resonance wavelength

The wafer-level measurement was conducted to record the centered resonance wavelengths on different chips. As we mentioned, the resonance wavelengths on different chips were influenced mostly by the wafer thickness variation. Consequently, the FP modulator shares an identical tendency with the micro-ring modulator for resonance wavelength shift. Here, we arranged the chip according to the actual chip mapping in the wafer and plotted the 3D bar chart to display the centered wavelength. To better demonstrate the wavelength distribution tendency on the whole wafer, we use λ-min(λ) as the Z-axis. Fig.S(a)(b) shows the centered resonance wavelength distribution for the FP and micro-ring modulators. Fig.S7(c)(d) shows the corresponding 2D flat mapping for the FP modulator and Micro-ring modulator for easier readability. It should be noted that the centered resonance wavelength is directly dependent on the SOI thickness, and a larger resonance wavelength indicates a thicker waveguide. Thus, we could evaluate the wafer thickness distribution according to the recorded resonance wavelength variation on different chips. The FP and micro-ring modulators reveal that the wafer has a thicker thickness in the middle. In other words, the wafer-level measurement for the centered resonance wavelength of the cavity structure is proven to be a method for wafer thickness evaluation.


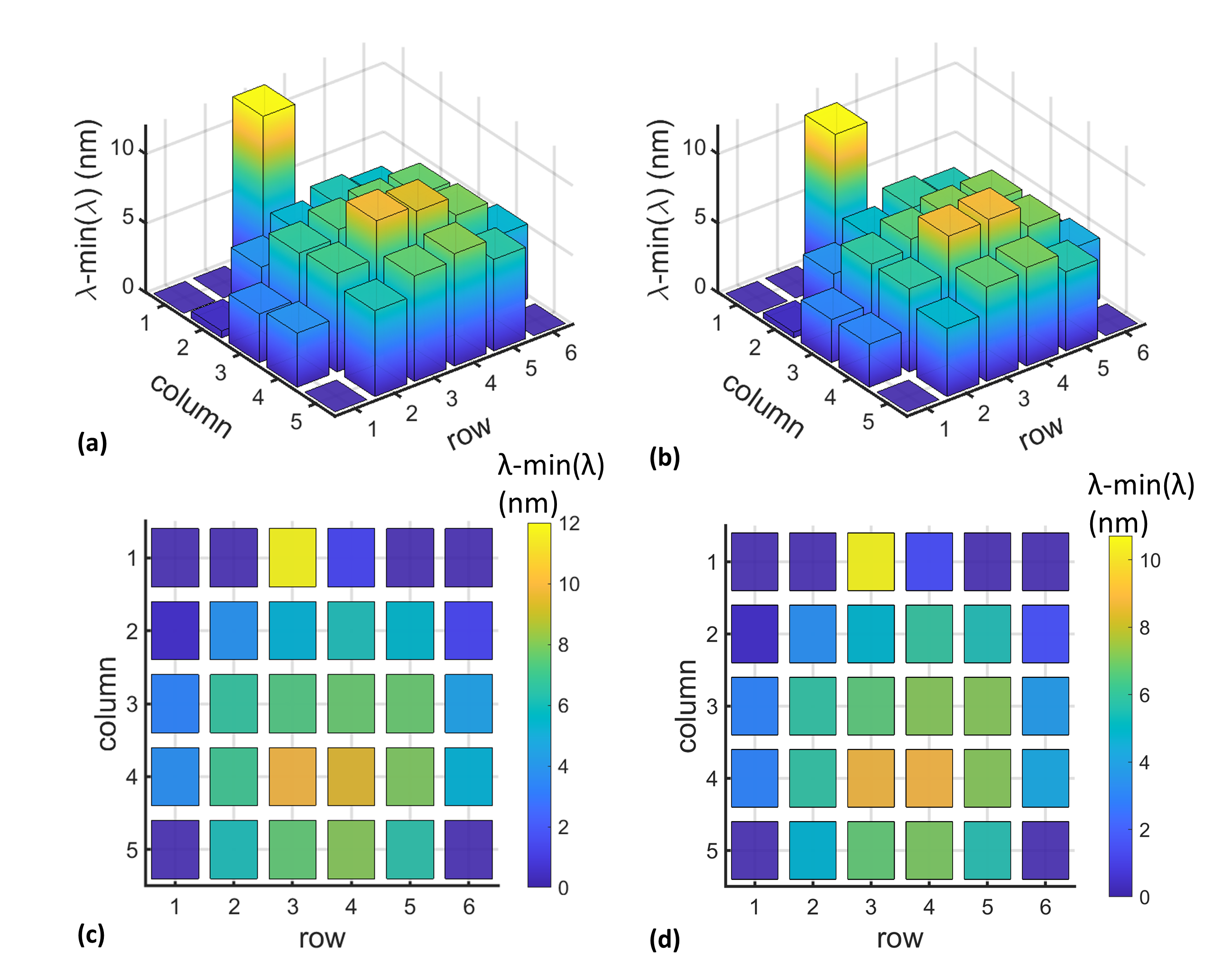


**Fig.S7.** The wafer-level centered resonance wavelength (λ-min(λ)) mapping for (a)FP modulator and (b)Micro-ring modulator; The 2D flat mapping for (c)FP modulator and (d)Micro-ring modulator.

References

[1] D. Dai *et al.*, “10‐Channel Mode (de)multiplexer with Dual Polarizations,” *Laser Photonics Rev.*, vol. 12, no. 1, p. 1700109, Jan. 2018, doi: 10.1002/lpor.201700109.

[2] O. Jafari, S. Zhalehpour, W. Shi, and S. LaRochelle, “Mode-conversion-based silicon photonic modulator loaded by a combination of lateral and interleaved p-n junctions,” *Photonics Res.*, vol. 9, no. 4, p. 471, Apr. 2021, doi: 10.1364/PRJ.414400.

[3] H. Jayatilleka, W. D. Sacher, and J. K. S. Poon, “Analytical Model and Fringing-Field Parasitics of Carrier-Depletion Silicon-on-Insulator Optical Modulation Diodes,” *IEEE Photonics J.*, vol. 5, no. 1, pp. 2200211–2200211, Feb. 2013, doi: 10.1109/JPHOT.2013.2240381.

[4] A. Hardy and W. Streifer, “Coupled mode theory of parallel waveguides,” *J. Light. Technol.*, vol. 3, no. 5, pp. 1135–1146, 1985, doi: 10.1109/JLT.1985.1074291.

[5] D. Liu and D. Dai, “Silicon-based polarization-insensitive optical filter with dual-gratings,” *Opt. Express*, vol. 27, no. 15, p. 20704, Jul. 2019, doi: 10.1364/OE.27.020704.

[6] D. Liu *et al.*, “High‐Performance Silicon Photonic Filter Using Subwavelength‐Structure Multimode Waveguide Gratings,” *Laser Photonics Rev.*, vol. 17, no. 12, p. 2300485, Dec. 2023, doi: 10.1002/lpor.202300485.

[7] S. Lin, M. Hammood, H. Yun, E. Luan, N. A. F. Jaeger, and L. Chrostowski, “Computational Lithography for Silicon Photonics Design,” *IEEE J. Sel. Top. Quantum Electron.*, vol. 26, no. 2, pp. 1–8, Mar. 2020, doi: 10.1109/JSTQE.2019.2958931.

[8] Y. Yuan *et al.*, “A 100 Gb/s PAM4 Two-Segment Silicon Microring Resonator Modulator Using a Standard Foundry Process,” *ACS Photonics*, vol. 9, no. 4, pp. 1165–1171, Apr. 2022, doi: 10.1021/acsphotonics.1c01389.
